# 

**Published:** 1875-01-01

**Authors:** 


*ORIGINAL COMMUNICATIONS:*

Fistula in Ano: Stricture of the Rectum; Operation. By F. K. Bailey, M.D. ........ 1

*TRANSLATIONS:*

Progress of Medical Science in Germany. By E. J. Doering, M.D. ........ 6

Gleanings from the French ........ 9

*EDITORIAL DEPARTMENT:*

The New Year ........ 11

Unusual Offer ........ 12

*SOCIETY REPORTS:*

Chicago Medical Society. Meeting of Dec. 7, 1874 ........ 13

Meeting of Dec. 21, 1874 ........ 20

Transactions of the Chicago Society of Physicians and Surgeons. Regular Meeting, Dec. 14, 1874 ........

